# Ameliorative Effects of Curcumin on Type 2 Diabetes Mellitus

**DOI:** 10.3390/molecules29122934

**Published:** 2024-06-20

**Authors:** Yujin Gu, Qun Niu, Qili Zhang, Yanfang Zhao

**Affiliations:** 1 School of Life Sciences and Medicine, Shandong University of Technology, Xincun West Road 266, Zhang Dian District, Zibo 255000, China; guyujin1725@126.com; 2 Institute of Xinhua Pharmaceutical, Shandong Xinhua Pharmaceutical Co., Ltd., Lutai Avenue 1, Gaoxin District, Zibo 255000, China; junyangniu123@163.com

**Keywords:** curcumin, type 2 diabetes mellitus, antidiabetic activity, molecular mechanisms

## Abstract

Type 2 diabetes mellitus (T2DM), a multifactorial and complicated metabolic disorder, is a growing public health problem. Numerous studies have indicated that bioactive compounds from herbal medicine have beneficial effects on T2DM prevention and treatment, owing to their numerous biological properties. Curcumin, the major curcuminoid of turmeric, is one of the most studied bioactive components of herbal supplements, and has a variety of biological activities. Clinical trials and preclinical research have recently produced compelling data to demonstrate the crucial functions of curcumin against T2DM via several routes. Accordingly, this review systematically summarizes the antidiabetic activity of curcumin, along with various mechanisms. Results showed that effectiveness of curcumin on T2DM is due to it being anti-inflammatory, anti-oxidant, antihyperglycemic, anti-apoptotic, and antihyperlipidemic, among other activities. In light of these results, curcumin may be a promising prevention/treatment choice for T2DM.

## 1. Introduction

Diabetes mellitus (DM), including type 1 (T1DM) and type 2 (T2DM), is a group of common metabolic endocrine diseases characterized by glucose and lipid metabolic disorder and hyperglycemia [1]. Among them, T2DM, the most prevalent form, accounts for more than 95% of all diabetic patients. Insulin resistance and insufficient compensatory insulin production are the two main contributors of T2DM [2,3]. The pathogenesis of T2DM is extremely complicated, and it is a polygenic genetic disease formed by the combined action of genetic and environmental factors. Firstly, genetic factors are the main factor. Secondly, the development of T2DM is related to the homeostasis of intestinal flora. Acquired factors also have an important impact on the development of T2DM. Obesity, sedentary lifestyle, physical inactivity, high-glycemic and low-fiber diet, vitamin deficiency, smoking, and alcohol consumption are complex factors that induce T2DM [4]. At present, the pathogenesis of T2DM is not completely clear. However, a growing body of research has revealed that T2DM is significantly influenced by a number of variables, including insulin resistance, inflammation, oxidative stress, lipid metabolism disorders, obesity, insulin secretion issues, intestinal flora, and others [5,6,7]. Moreover, T2DM is associated with a series of complications, including microvascular complications, macrovascular complications, renal complications, cardiac complications, and diabetic gastroenteropathy, which can significantly lower the patient’s quality of life and even lead to death [8,9]. Although recent studies have given a new look to the understanding of T2DM, currently available treatments can only temporarily reduce blood glucose levels but cannot completely prevent the development of T2DM and its complications. Most antidiabetic drugs have side effects, such as gastrointestinal symptoms, heart failure, weight gain, edema, impaired kidney function pancreatitis, and genital infections, which become another burden on patients [10]. Therefore, new antidiabetic agents with less side effects are necessary.

Natural medicines have many advantages over traditional medicines, including fewer side effects, lower long-term toxicity, and varied bioavailability. Numerous studies have indicated that bioactive compounds from herbal medicine, such as polyphenols, flavonoids, and alkaloids, have beneficial effects on T2DM prevention and treatment, by improving glucose tolerance, insulin resistance, and other related mechanisms [11,12].

Curcumin, a natural polyphenol derived from the rhizome of Curcuma longa (turmeric), which has been widely used in cosmetics, food, and pharmaceutical industries, has gained growing interest in the last years for its pharmacological activities. Different studies demonstrated that curcumin has anti-oxidant, anti-inflammatory, antimicrobial, anti-atherosclerotic, nephroprotective, anticancer, hepatoprotective, immunomodulatory, antidiabetic, and antirheumatic effects, but with no toxicity [13,14,15,16,17,18]. Numerous studies demonstrated that curcumin could improve insulin resistance, regulate blood lipid metabolism, decrease glucose and insulin levels, reduce the release of inflammatory factors, inhibit oxidative stress, and regulate gut microbiota in patients with T2DM [19,20,21,22]. Given the above, this review aimed to summarize the effects of curcumin on T2DM through anti-inflammatory, free radical scavenging, upregulation of antioxidant enzymes, regulation of blood lipid metabolism, and other pathways.

## 2. Review Methodology

With the Chinese and English words of “diabetes mellitus”, “type 2 diabetes mellitus”, “curcumin”, “curcuminoid”, “antidiabetic activity”, and “molecular mechanisms” as keywords, we combined and searched the relevant literature published in PubMed, Web of Science, ScienceDirect, China National Knowledge Internet (CNKI), Wanfang, and Weipu. Publications from 2000 onwards were taken into consideration, with a preferential emphasis on larger research carried out in the last five years (2019–2023). References older than five years are only taken into account only when a statement is important in the context and needs to be emphasized. The advancements in the studies designed to investigate the effects of curcumin on T2DM are summarized in this paper, which will provide a better understanding about the antidiabetic activity of curcumin.

## 3. Properties of Curcumin

### 3.1. Physical and Chemical Properties of Curcumin

**Figure 1:**
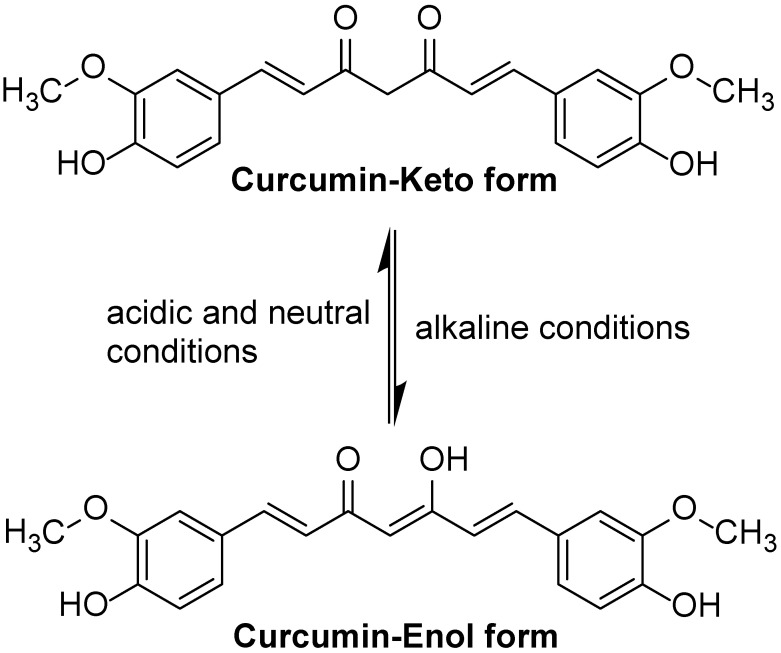
The keto form and enol form structures of curcumin.

The solubility and stability of curcumin depend on its environmental pH. Curcumin is very insoluble in water, but soluble in organic solvents. In acidic to neutral pH, curcumin is relatively stable, whereas in alkaline pH, curcumin is unstable and easily degradable. The degradation products that have been identified include ferulic acid, ferulic aldehyde, and vanillin. Similarly, curcumin photodegrades into smaller phenols when exposed to light. However, the degradation of curcumin was markedly inhibited when surfactants, albumins, cell culture medium, and biological fluids were present. Moreover, the degradation proceeds mainly through the keto–enol link. In addition, studies indicated that curcumin’s overall activity may be attributed to degradation products such ferulic acid and vanillin, which have better bioavailability and antioxidant activity [23]. Under acidic and neutral conditions, curcumin exists in keto form. Because curcumin’s keto form has a highly active carbon atom in its heptadienone connection between the two methoxyphenol rings, curcumin may contribute a H atom at such pHs. Under alkaline conditions, it exists mainly in the enol form, where curcumin donates electrons in a mechanism that reflects its antioxidant scavenging activities (Figure 1) [24]. The enol form of curcumin has three ionizable protons, corresponding to the enolic group and two phenolic groups. Of the three dissociable protons, the enolic proton is the most acidic, which has significant effects on curcumin’s radical-scavenging mechanisms related to proton transfer and dissociation [25].

### 3.2. Pharmacokinetics and Toxicology of Curcumin

**Figure 2:**
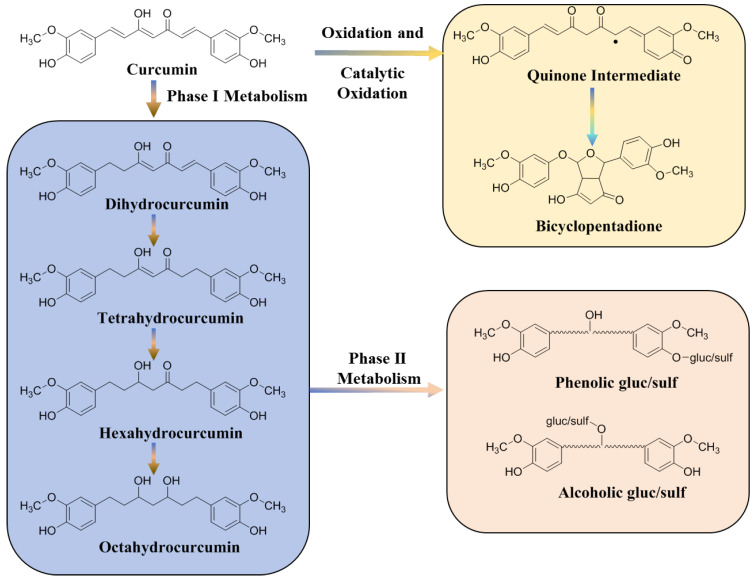
Metabolic pathways of curcumin in vivo. The dot represents an electron.

Numerous investigations have demonstrated that curcumin has low membrane permeability and little absorption in the gastrointestinal tract following oral treatment. Additionally, the low absorption of curcumin might be attributed to its hepatoenteric first pass effect. The rate of absorption is also determined by the delivery route. For instance, compared to intravenous or oral modes of delivery, the intraperitoneal route demonstrated higher levels of curcumin in plasma [26,27,28]. In addition, the highly reactive structure of curcumin leads to its easy degradation, which causes poor distribution to specific locations [29]. Curcumin is metabolized rapidly in the body and mainly undergoes phase I reduction metabolism, phase II binding metabolism, auto-oxidation, and intracellular catalytic oxidation metabolism (Figure 2). Following a step-by-step hydrogenation process, dihydrocurcumin, tetrahydrocurcumin, and hexahydrocurcumin are the primary metabolites of curcumin I phase reduction metabolism. Since the phase I metabolites have the structure of phenolic and alcohol hydroxyl groups, the binding reaction of gluconaldehyde and sulfuric acid occurs in phase II metabolism. The final metabolites of curcumin are mainly glycosylated products and relatively few sulfonated products [30]. Moreover, all these metabolites have been synthesized and tested for possible activity. Because there are conflicting views in the literature about the activity of these metabolites, it is currently unclear if these metabolites are more active than curcumin. Tetrahydrocurcumin has been found to be less anticancer active and a better antioxidant than curcumin thus far [31]. As stated above, curcumin has low penetration, extensive metabolism, low bioavailability and targeting efficacy, which are the main limiting factors for its therapeutic application. Despite its low bioavailability, curcumin still has a wide range of pharmacological activities.

Long-term studies have shown that curcumin is safe and protective when used in the diet. The United States Food and Drug Administration considers curcumin as to be a ‘generally recognized as safe’ product, and clinical trials have shown that it has strong tolerability and safety profiles at doses ranging from 4000 to 8000 mg [32]. In phase I clinical studies, curcumin with doses up to 3600–8000 mg daily for 4 months did not result in discernible toxicities, except mild nausea and diarrhea [33].

## 4. Curcumin and T2DM

### 4.1. Curcumin Alleviates Inflammation in T2DM by Inhibiting the Production of Pro-Inflammatory Mediators

Inflammation is one of the primary pathogenic factors of T2DM and is crucial to the emergence and progression of insulin resistance, as well as the rise in blood glucose levels. Conversely, the presence of hyperglycemia might promote insulin resistance and long-term complications [34]. Through various transcription factor-mediated molecular pathways and oxidative stress, inflammatory responses can activate various pro-inflammatory mediators, especially cytokines, chemokines, and adipokines, such as interleukin-1β (IL-1β), interleukin-6 (IL-6), interleukin-18 (IL-18), tumor necrosis factor α (TNF-α), monocyte chemoattractant protein-1 (MCP-1), and transforming growth factor-β1(TGF-β1). These inflammatory mediators reduce tissue insulin-mediated glucose uptake and insulin signal transduction by activating Jun NH2-terminal kinase (JNK) and nuclear factor kappa-B (NF-κB) pathways. In addition, the activation of JNK and NF-κB pathways also promotes the upregulation of various pro-inflammatory mediators such as TNF-α and IL-6, which further aggravates insulin resistance and accelerates the occurrence and development of T2DM [35,36,37].

**Figure 3:**
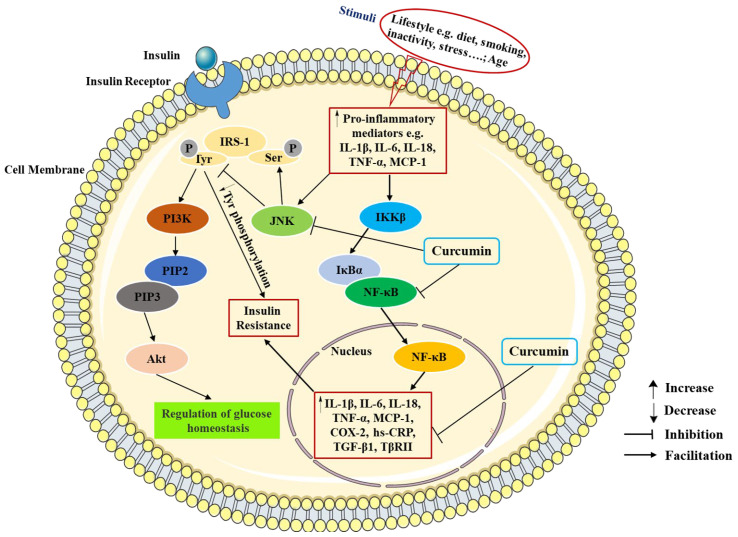
The anti-inflammatory effects of curcumin in T2DM. IRS-1, insulin receptor substrate-1; ser, serine; tyr, tyrosine; PI3K, phosphoinositide 3-kinase; PIP2, phosphatidylinositol-4,5-bisphosphate; PIP3, phosphatidylinositol-3,4,5-triphosphate; Akt, protein kinase B; JNK, Jun NH2-terminal kinase; IKKβ, IκB kinase-β; IκBα, inhibitor kappa B-α; NF-κB, nuclear factor kappa B; TNF-α, tumor necrosis factor-α; IL-1β, interleukin-1β; IL-6, interleukin-6; IL-18, interleukin-18; MCP-1, monocyte chemotactic protein-1; hs-CRP, high sensitivity C-reactive protein; COX-2, cyclooxygenase-2; TGF-β1, transforming growth factor-β1; TβRII, type II TGF-β.

Several studies have indicated that curcumin exerts a protective effect against diabetes through the inhibition of inflammation (Table 1). The experiments revealed that curcumin treatment reduced the serum inflammatory factor levels of glycosylated hemoglobin (HbA1c), MCP-1, IL-6, and TNF-α in diabetic rats through suppressing the NF-κB pathway [38]. Abo-Salem et al. demonstrated that curcumin dramatically decreased IL-6 and TNF-α secretion in streptozotocin (STZ)-induced diabetic rats with heart injury [39]. A similar study suggested that curcumin significantly suppressed MCP-1, IL-1β, TNF-α, IL-6, and cyclooxygenase-2 (COX-2) production in adipocytes [40]. Guo et al. demonstrated that curcumin inhibited TGF-β1 and type II TGF-β (TβRII) production and blocked the non-canonical adenosine monophosphate-activated protein kinase/p38 mitogen-activated protein kinase (AMPK/p38 MAPK) pathway in diabetic rat hearts [41]. A study showed that the curcumin and its analog alleviated diabetes-induced damages by regulating inflammation in the brain of diabetic rats [42]. Another study revealed that the administration of curcumin decreased serum levels of TNF-α and increased the serum level of adiponectin [43]. Adibian et al. demonstrated that curcumin supplementation could significantly reduce the concentration of high sensitivity C-reactive protein (hs-CRP) and increase the concentration of adiponectin in patients with T2DM [44]. Furthermore, curcumin could inhibit the JNK phosphorylation to prevent apoptotic and inflammatory processes in diabetic cardiomyopathy [45]. In short, these data show that curcumin supplementation fosters anti-inflammatory factor production, such as adiponectin, and reduces the pro-inflammatory cytokine production, such as TNF-α, IL-6, IL-1β, and MCP-1 in T2DM subjects. The anti-inflammatory effects of curcumin on T2DM are shown in Figure 3.

### 4.2. Curcumin Reduces the Oxidative Stress in T2DM

Many studies have shown that oxidative stress is closely related to the pathogenesis of T2DM [46,47]. Hyperglycemia can increase the production of free radicals, which further leads to the occurrence of oxidative stress. In turn, elevated production of free radicals can damage the antioxidant defense system and lead to the generation of glucose-derived advanced glycosylation end products (AGEs). The accumulation of AGEs in the body can induce oxidative damage of cell membranes, cell function damage, enhanced lipid peroxidation, and various complications of T2DM. All of these events eventually lead to pancreatic islet β-cell dysfunction, insufficient insulin secretion and insulin resistance, which aggravate the development of T2DM and its complications [48,49,50]. Generally, the activities of antioxidant enzymes such as superoxide dismutase (SOD), catalase (CAT), and glutathione peroxidase (GSH-Px) reflect the status of oxidative stress. In addition, the levels of malondialdehyde (MDA), a product of lipid peroxidation, were also used to reflect the level of oxidative stress. Moreover, nitric oxide (NO), a product of inducible nitric oxidesynthase (iNOS), could exacerbate oxidative stress [49,51].

**Figure 4:**
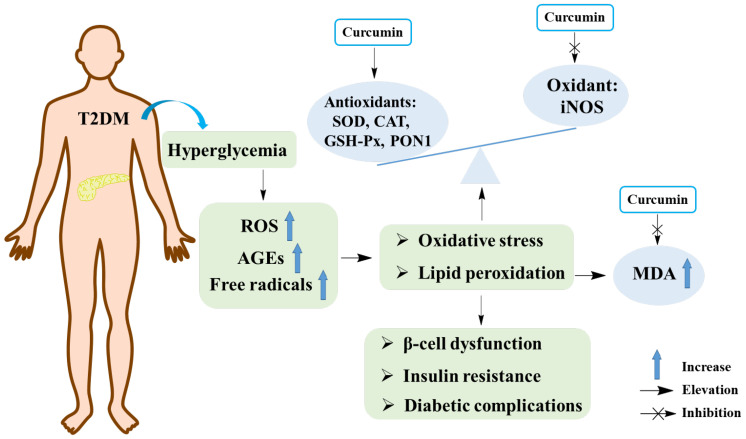
The anti-oxidant effects of curcumin in T2DM. SOD, superoxide dismutase; CAT, catalase; GSH-px, glutathione peroxidase; iNOS, inducible nitric oxide synthase; MDA, malondialdehyde; ROS, reactive oxygen species; PON1, paraoxonase-1; AGEs, advanced glycosylation end products.

Studies indicated that curcumin is a natural antioxidant. A study elucidated that curcumin decreased the amount of MDA and increased the level of SOD, as well as diminished the ratio of apoptosis in alloxan (AXN)-treated pancreatic islet cells, suggesting that curcumin could be a potential compound for protecting pancreatic islet cells and treating T2DM [52]. Results from a meta-analysis showed that curcumin had antioxidant effect by lowering MDA levels and increasing SOD activity [53]. Shafabakhsh et al. reported that curcumin oral administration for 12 weeks (1000 mg/day) in patients with T2DM could improve the values of total antioxidant capacity (TAC), glutathione (GSH), MDA, and the gene expression of peroxisome proliferator-activated receptor gamma (PPAR-γ) [54]. In addition, curcumin could decrease lipid peroxidation, likely by increasing ATPase activity, restoring oxygen consumption and NO synthesis in the liver and kidneys of diabetic mice, which suggested that curcumin could be a better substitute to prevent and/or treat oxidative stress and mitochondrial dysfunction during obesity and diabetes [55]. In a randomized double-blind placebo-controlled trial, the use of curcuminoids (1000 mg/day co-administered with piperine 10 mg/day) dramatically lowered the level of MDA in T2DM patients while considerably increasing the activities of TAC and SOD [56]. Additionally, it was demonstrated that curcumin could not only elevate the activities of SOD, CAT, and paraoxonase-1 (PON1), but also increase the amounts of AGEs and detoxification system components (AGE-R1 receptor and glyoxalase-1) in STZ-induced diabetic rats [57]. The anti-oxidation effects of curcumin on T2DM are exhibited in Figure 4.

### 4.3. Curcumin Regulates Lipid Metabolism in T2DM

In addition to hyperglycemia, T2DM patients are often accompanied by lipid metabolism disorder. Elevated circulating levels of lipids and excessive deposition of fat in non-adipose tissues, such as muscle and liver, are known as lipotoxicity [58]. During the onset and progression of T2DM, lipotoxicity can contribute to or exacerbate insulin resistance, pancreatic β-cell dysfunction, and death [59]. Furthermore, studies revealed that lipotoxicity in β-cells triggers different stress pathways, especially the endoplasmic reticulum (ER) stress and oxidative stress, ultimately leading to β-cells dysfunction and death [60]. In addition, the dysregulation of AMPK and downstream effectors play an important role in the pathogenesis of hepatic steatosis, dyslipidemia, and insulin resistance [6,61].

In a double-blind randomized clinical trial, results indicated that curcumin treatment may diminish diabetic complications by reducing the serum levels of triglycerides (TGs) in patients with T2DM [44]. A study argued that curcumin improves insulin resistance and glucose homeostasis in db/db mice by regulating lipid metabolism. Results showed that curcumin significantly lowered plasma free fatty acids (FFAs), total cholesterol (TC), and TGs concentrations in type 2 diabetic mice. Moreover, curcumin could alter the activities of hepatic fatty acid synthase (FAS), β-oxidation, carnitine palmitoyltransferase (CPT), 3-hydroxy-3-methylglutaryl coenzyme (HMG-CoA) reductase, and acyl-CoA:cholesterol acyltransferase (ACAT) in db/db mice. Furthermore, curcumin increased the lipoprotein lipase (LPL) activity of skeletal muscles in db/db mice [62]. Another study inferred that the effect of curcumin on insulin resistance might be correlated with the decreases of FFAs and low-density lipoprotein (LDL) levels in T2DM rats [63]. Similarly, Belhan et al. revealed that curcumin significantly ameliorated lipid profiles in STZ-induced diabetic rats [64]. In addition, results showed that curcumin could inhibit renal lipid accumulation and oxidative stress through AMPK and nuclear factor (erythroid-derived 2)-like 2 (Nrf2) signaling pathways in a rat model of type 2 diabetic nephropathy [65]. Curcumin and nanocurcumin treatment significantly decreased insulin resistance and serum levels of fasting blood sugar (FBS), apelin, TC, TGs, LDL, and very low-density lipoproteins (VLDL), as well as increasing the high-density lipoprotein (HDL) levels in diabetic rats. Moreover, the nanocurcumin was more effective in alleviating the lipid profile than that of curcumin [66]. A study performed by Devadasu et al. demonstrated that curcumin nanoparticulate administration significantly reduced plasma TGs and TC levels, whilst it increased HDL in STZ-induced diabetic rats [67]. In a randomized, double-blind placebo-controlled phase 2 clinical trial, results indicated that curcumin and zinc co-supplementation along with a weight loss diet could improve lipid profiles, including TGs, LDL, HDL, non-HDL, and HDL to LDL ratio in patients with prediabetes [68]. Consistent with these results, Panahi et al. reported that curcuminoids treatment could reduce serum levels of TC, non-HDL, and Lp(a), as well as elevate HDL levels in patients with T2DM [69]. Moreover, curcumin ameliorated fat accumulation, serum lipid levels, and insulin sensitivity through regulating sterol regulatory element-binding proteins (SREBPs) target genes, and metabolism-associated genes in the liver or adipose tissues in high fat diet-induced obese mice with T2DM [70]. Given the above, the ameliorative effects of curcumin on T2DM may be related to its regulation of lipotoxicity.

### 4.4. Curcumin Lowers Blood Glucose Levels and Improves Insulin Resistance in T2DM

Chronic hyperglycemia and AGEs can lead to tissue oxidative stress and pancreatic β-cell glucotoxicity, causing loss of homeostasis, which further aggravates hyperglycemia [71]. Therefore, maintaining blood glucose homeostasis might be an effective antidiabetic intervention. Furthermore, the activation of glucose transporter 4 (GLUT4) and various enzymes such as glucose 6-phosphate (G6P), phosphoenolpyruvate carboxykinase (PEPCK), glycogen synthase (GS), and hexokinase (HK) are involved in glucose transport and metabolism. The phosphoinositide 3-kinase (PI3K)/protein kinase B (Akt) pathway and the activity of AMPK play an essential role in regulating glucose metabolism and energy homeostasis [72].

Numerous investigations revealed that curcumin is an effective antihyperglycemia agent. In a clinical trial, the findings detected that curcumin could improve insulin resistance, lower blood glucose levels, and reduce circulating glycogen synthase kinase-3 beta (GSK-3β), as well as islet amyloid polypeptide (IAPP) [73]. Another clinical trial found that the curcumin administration significantly reduced fasting blood glucose (FBG), HbA1c, and estimated average glucose (eAG) levels [74]. Algul et al. demonstrated that oral curcumin administration improved FBG, significantly up-regulated GLUT4 gene expression, and improved nesfatin-1 [75]. Chang et al. revealed that curcumin enhanced insulin sensitivity and improved glucose intolerance in addition to lowering the FBG and increasing GLUT4 gene expression [76,77]. Similar findings came from additional research [78]. PPARγ play a very important role in the regulation of glucose metabolism. Numerous investigations have demonstrated that curcumin-induced PPARγ activation can inhibit the surface expression of glucose transporter 2 (GLUT2) and AGEs accumulation [47,79,80]. Chuengsamarn et al. indicated that curcumin ameliorated the overall performance of β-cells with higher homeostasis model assessment (HOMA-β) and lower C-reactive protein (CRP) [81]. Additionally, studies revealed that curcumin could prevent hyperglycemia by promoting insulin secretion, improving β-cell function and inhibiting β-cell apoptosis [82,83,84]. With regard to the anti-hyperglycemic activity, curcumin presents a viable option for T2DM prevention or treatment.

### 4.5. Other Effects of Curcumin on T2DM

**Figure 5:**
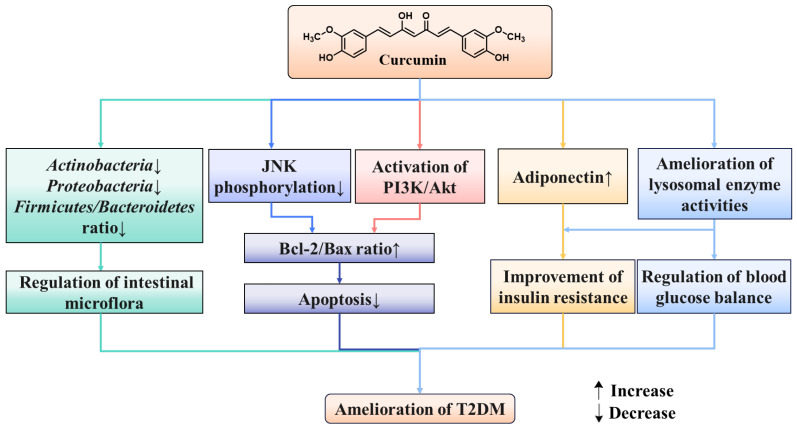
Other effects of curcumin in T2DM.

It is well known that gut microbiota plays a key role in human disease progression. In recent years, high concentrations of curcumin have been detected in the gastrointestinal tract after oral administration, indicating that it can directly interact with the gut microbiota and exert regulatory effects [85]. It is worth noting that two different phenomena have emerged in the interaction between curcumin and the microbiota; the regulation of curcumin on the gut microbiota and the biotransformation of curcumin by the gut microbiota, both of which may be crucial for the activity of curcumin. Several studies have confirmed that insulin resistance and the onset of T2DM are closely associated with gut microbiome disorders [86,87]. An analysis indicated that tetrahydrocurcumin alleviated the blood glucose level, up-regulated the expression of pancreatic glucagon-like peptide-1(GLP-1), and promoted the secretion of insulin by reducing the relative abundance of *Actinobacteria*, *Proteobacteria*, and *Firmicutes*/*Bacteroidetes* ratio in diabetic rats [88]. Ren et al. demonstrated that curcumin reduced high glucose-induced apoptosis in cardiomyocytes through the inhibition of JNK phosphorylation in the diabetic heart [89]. Wang et al. investigated that curcumin analog supplement significantly reversed the diabetes-induced cardiac cells apoptosis by decreasing the anti-apoptotic protein (Bcl-2) and improving the breakdown of pro-apoptotic protein (Bax) and caspase-3 in diabetic mice [90]. A curcumin complement effectively suppressed the elevated Bax/Bcl-2 ratio and ameliorated glucose-induced cardiomyocyte apoptosis in neonatal rat cardiomyocytes, accompanied by increased Akt and GSK-3 phosphorylation [91]. An investigation obtained that curcumin could mitigate T2DM by increasing adiponectin levels and decreasing C peptide levels along with insulin resistance [81]. Furthermore, curcumin could regulate lysosomal enzyme activities in diabetes [92]. The other effects of curcumin on T2DM are exhibited in Figure 5.

## 5. Conclusions and Perspectives

In view of the increasing incidence rate of T2DM worldwide, it is very important to find effective drugs for T2DM. Curcumin, the major curcuminoid of turmeric, has various positive benefits on T2DM. Numerous studies, including animal and clinical studies, have provided strong evidence to support curcumin’s crucial role in T2DM prevention due to its anti-inflammatory, anti-oxidant, antihyperglycemic, anti-apoptotic, antihyperlipidemic, and other actions.

The utilization of curcumin to treat T2DM has some limitations like poor solubility, low instability, low bioavailability, low penetration, extensive metabolism, and targeting efficacy [93]. Among these, the main hurdle of curcumin therapeutic application in T2DM is its poor bioavailability. Some methods were designed to enhance the solubility, durability, and bioavailability of curcumin. For instance, the bioavailability of curcumin can be improved by combining with piperine and other bioavailability enhancers or constructing curcumin complexes [32]. In addition, the emergence of nanobiotechnology, such as liposomes, microemulsion, hydrophilic prodrugs, solid nanoparticles, polymer micelles and nanogels, has opened up broad opportunities for exploring and expanding the application of curcumin in the medical field. Compared with regular curcumin, nanocurcumin has better half-life, absorption capacity, better drug delivery ability and high bioavailability [94]. Therefore, in addition to nanobiotechnology, new drug delivery systems should be investigated and applied to enhance the clinical benefits of curcumin to T2DM.

The various activities and biological activities of curcumin depend on its excited xtate intramolecular hydrogen transfer (ESIHT) process. Curcumin interacts with a number of biomolecules, such as proteins, nucleic acids, membranes, through non-covalent and covalent binding. Non-covalent interactions in curcumin are caused by hydrogen bonding, hydrophobicity, and the flexibility of the linker group, which are derived from the aromatic and tautomeric structures. The α, β-unsaturated β-diketone moiety covalently interacts with protein thiols, through the Michael reaction. The formation of chelates between β-diketone groups and transition metals can reduce metal-induced toxicity, and some metal complexes exhibit better antioxidant activity as enzyme mimetics [31]. It has been discovered that the glycosylated derivatives increase curcumin’s water solubility. Curcumin’s metabolic stability may be enhanced by derivatizing the diketone moiety with functionalized pyrazole, hydrazine, semicarbazone, thiosemicarbazone, and thiophene moieties, among other modifications [95]. New analogues with improved activity may be developed with modifications on specific functional groups of curcumin. Hence, synthesis of curcumin analogues is also one of the effective methods to enhance its biological activity.

In addition, the antidiabetic activity of curcumin is mainly carried out in animal and cell models and there are few clinical data. Hence, comprehensive research on the hypoglycemic effect of curcumin requires extensive clinical trials. Since it is commonly recognized that different pharmacological dosages have varying effects, curcumin dosage should be a focus of future research. It is worth noting that challenges related to the utilization of plant medicines include insufficient bioavailability and inconsistent commercially available product quality. Therefore, it is imperative to develop optimized formulas and standardized extraction methods. Furthermore, it is necessary to conduct a comprehensive investigation into the safety, long-term effects, and potential drug interactions of curcumin.

As stated above, curcumin exhibits good hypoglycemic action and is well taken at high dosages without adverse effects. It is therefore a potential approach for T2DM treatment or prevention.

## Figures and Tables

**Table 1:** The anti-inflammatory effects of curcumin in type 2 diabetes mellitus (T2DM).

Diabetic Model	Concentration/Dosage	Duration	Effects	Ref.
High glucose-treated U937 monocytes	0.01–1 μM	24 h	↓ MCP-1, IL-6, HbA1c, TNF-α and lipid peroxidation; ↓ Blood glucose; ↓ Oxidative stress	[38]
Streptozotocin-induced diabetic rats	100 mg/kg BW/day	7 weeks		
Streptozotocin-induced diabetic rats	200 mg/kg BW/day	6 weeks	↓TNF-α, IL-6	[39]
Adipocytes	20 µM	62 h	↓ MCP-1, IL-1β, TNF-α, IL-6 and COX-2	[40]
High glucose-treated human cardiac fibroblasts	25 μM	24 h	↓ TGF-β1, TβRII, Smad2/3 phosphorylation and high glucose-induced AMPK/p38 MAPK activation; ↓ Cardiac fibrosis in the fibroblasts	[41]
Streptozotocin-induced diabetic rats	300 mg/kg BW/day	16 weeks		
Streptozotocin-induced diabetic rats	20 mg/kg BW/day	8 weeks	↓ Blood glucose; ↓ NF-κB p65, TNF-α and COX-2; ↑ Activity of SOD; ↓ MDA	[42]
50 patients with type 2 diabetes	1000 mg/day co-administered with piperine 10 mg/day	12 weeks	↑ Adiponectin levels; ↓ Leptin levels, leptin/adiponectin ratio; ↓ TNF-α	[43]
22 patients with type 2 diabetes	1500 mg/day	10 weeks	↓ hs-CRP; ↑ Serum concentration of adiponectin	[44]
High glucose-stimulated primary cultures of neonatal rat cardiomyocytes and H9c2 cells	2.5, 5, or 10 µM	2 h	↓ TNF-α expression; ↓ TNF-α, IL-1β, IL-6, IL-12 mRNA transcription; ↓ JNK phosphorylation; ↓ activation of NF-kB;	[45]
Streptozotocin-induced diabetic rats	5 mg/kg once every 2 days	12 weeks		

Abbreviations: ↑ Increase; ↓ Decrease; MCP-1, monocyte chemoattractant protein-1; IL-6, interleukin-6; HbA1c, glycosylated hemoglobin; TNF-α, tumor necrosis factor α; IL-1β, interleukin-1β; COX-2, cyclooxygenase-2; TGF-β1, transforming growth factor-β1; TβRII, type II TGF-β; AMPK/p38 MAPK, adenosine monophosphate-activated protein kinase/p38 mitogen-activated protein kinase; NF-κB, nuclear transcriptor factor kappa B; SOD, superoxide dismutase; MDA, malondialdehyde; hs-CRP, high sensitivity C-reactive protein; JNK, Jun NH2-terminal kinase.

## Data Availability

Not applicable.
